# Analysis of Psychological Symptoms Following Disclosure of Amyloid–Positron Emission Tomography Imaging Results to Adults With Subjective Cognitive Decline

**DOI:** 10.1001/jamanetworkopen.2022.50921

**Published:** 2023-01-13

**Authors:** Camilla Caprioglio, Federica Ribaldi, Leonie N. C. Visser, Carolina Minguillon, Lyduine E. Collij, Oriol Grau-Rivera, Philip Zeyen, José Luis Molinuevo, Juan Domingo Gispert, Valentina Garibotto, Christian Moro, Zuzana Walker, Paul Edison, Jean-François Demonet, Frederik Barkhof, Philip Scheltens, Isadora Lopes Alves, Rossella Gismondi, Gill Farrar, Andrew W. Stephens, Frank Jessen, Giovanni B. Frisoni, Daniele Altomare

**Affiliations:** 1 Laboratory of Neuroimaging of Aging (LANVIE), University of Geneva, Geneva, Switzerland; 2 Geneva Memory Center, Geneva University Hospitals, Geneva, Switzerland; 3 Division of Clinical Geriatrics, Department of Neurobiology, Care Sciences and Society, Center for Alzheimer Research, Karolinska Institutet, Stockholm/Solna, Sweden; 4 Department of Medical Psychology, Amsterdam Public Health Research Institute, Amsterdam University Medical Centers (UMC)–Location VUmc, Amsterdam, the Netherlands; 5 Barcelonaßeta Brain Research Center (BBRC), Pasqual Maragall Foundation, Barcelona, Spain; 6 IMIM (Hospital del Mar Medical Research Institute), Barcelona, Spain; 7 Centro de Investigación Biomédica en Red de Fragilidad y Envejecimiento Saludable (CIBERFES), Madrid, Spain; 8 Department of Radiology and Nuclear Medicine, Amsterdam University Medical Centers (UMC)–Location VUmc, Amsterdam, the Netherlands; 9 Department of Psychiatry, Faculty of Medicine and University Hospital Cologne, University of Cologne, Cologne, Germany; 10 H. Lundbeck A/S, Denmark; 11 Centro de Investigación Biomédica en Red Bioingeniería, Biomateriales y Nanomedicina, (CIBER-BBN), Barcelona, Spain; 12 Laboratory of Neuroimaging and Innovative Molecular Tracers (NIMTlab), Geneva University Neurocenter and Faculty of Medicine, University of Geneva, Geneva, Switzerland; 13 Division of Nuclear Medicine and Molecular Imaging, Geneva University Hospitals, Geneva, Switzerland; 14 Center for Biomedical Imaging (CIBM), Geneva, Switzerland; 15 Division of Psychiatry, University College London, London, United Kingdom; 16 Margaret’s Hospital, Essex Partnership University NHS Foundation Trust, Essex, United Kingdom; 17 Division of Neurology, Department of Brain Sciences, Imperial College London, United Kingdom; 18 Leenaards Memory Center, Lausanne University Hospital (CHUV), Lausanne, Switzerland; 19 Queen Square Institute of Neurology and Centre for Medical Image Computing, University College London, London, United Kingdom; 20 Alzheimer Center, Department of Neurology, Amsterdam University Medical Centers (UMC)–Location VUmc, Amsterdam, the Netherlands; 21 Life Molecular Imaging, Berlin, Germany; 22 GE Healthcare, Amersham, United Kingdom; 23 German Center for Neurodegenerative Diseases (DZNE), Bonn-Cologne, Germany; 24 Excellence Cluster Cellular Stress Responses in Aging-Related Diseases (CECAD), Medical Faculty, University of Cologne, Germany

## Abstract

**Importance:**

Individuals who are amyloid-positive with subjective cognitive decline and clinical features increasing the likelihood of preclinical Alzheimer disease (SCD+) are at higher risk of developing dementia. Some individuals with SCD+ undergo amyloid-positron emission tomography (PET) as part of research studies and frequently wish to know their amyloid status; however, the disclosure of a positive amyloid-PET result might have psychological risks.

**Objective:**

To assess the psychological outcomes of the amyloid-PET result disclosure in individuals with SCD+ and explore which variables are associated with a safer disclosure in individuals who are amyloid positive.

**Design, Setting, and Participants:**

This prospective, multicenter study was conducted as part of The Amyloid Imaging to Prevent Alzheimer Disease Diagnostic and Patient Management Study (AMYPAD-DPMS) (recruitment period: from April 2018 to October 2020). The setting was 5 European memory clinics, and participants included patients with SCD+ who underwent amyloid-PET. Statistical analysis was performed from July to October 2022.

**Exposures:**

Disclosure of amyloid-PET result.

**Main Outcomes and Measures:**

Psychological outcomes were defined as (1) disclosure related distress, assessed using the Impact of Event Scale–Revised (IES-R; scores of at least 33 indicate probable presence of posttraumatic stress disorder [PTSD]); and (2) anxiety and depression, assessed using the Hospital Anxiety and Depression scale (HADS; scores of at least 15 indicate probable presence of severe mood disorder symptoms).

**Results:**

After disclosure, 27 patients with amyloid-positive SCD+ (median [IQR] age, 70 [66-74] years; gender: 14 men [52%]; median [IQR] education: 15 [13 to 17] years, median [IQR] Mini-Mental State Examination [MMSE] score, 29 [28 to 30]) had higher median (IQR) IES-R total score (10 [2 to 14] vs 0 [0 to 2]; *P* < .001), IES-R avoidance (0.00 [0.00 to 0.69] vs 0.00 [0.00 to 0.00]; *P* < .001), IES-R intrusions (0.50 [0.13 to 0.75] vs 0.00 [0.00 to 0.25]; *P* < .001), and IES-R hyperarousal (0.33 [0.00 to 0.67] vs 0.00 [0.00 to 0.00]; *P* < .001) scores than the 78 patients who were amyloid-negative (median [IQR], age, 67 [64 to 74] years, 45 men [58%], median [IQR] education: 15 [12 to 17] years, median [IQR] MMSE score: 29 [28 to 30]). There were no observed differences between amyloid-positive and amyloid-negative patients in the median (IQR) HADS Anxiety (–1.0 [–3.0 to 1.8] vs –2.0 [–4.8 to 1.0]; *P* = .06) and Depression (–1.0 [–2.0 to 0.0] vs –1.0 [–3.0 to 0.0]; *P* = .46) deltas (score after disclosure – scores at baseline). In patients with amyloid-positive SCD+, despite the small sample size, higher education was associated with lower disclosure-related distress (ρ = –0.43; *P* = .02) whereas the presence of study partner was associated with higher disclosure-related distress (*W* = 7.5; *P* = .03). No participants with amyloid-positive SCD+ showed probable presence of PTSD or severe anxiety or depression symptoms at follow-up.

**Conclusions and Relevance:**

The disclosure of a positive amyloid-PET result to patients with SCD+ was associated with a bigger psychological change, yet such change did not reach the threshold for clinical concern.

## Key Points

**Question:**

What are the psychological symptoms following disclosure of a positive amyloid–positron emission tomography (PET) imaging result on patients with subjective cognitive decline?

**Findings:**

This study of 105 patients found that the disclosure of a positive amyloid-PET result was associated with a greater psychological changes, yet such changes did not reach the threshold for clinical concern.

**Meaning:**

These findings suggest that the disclosure of a positive amyloid-PET result in patients with subjective cognitive decline was not associated with clinically meaningful psychological risk.

## Teaser

This study investigates the psychological outcome of the amyloid-positron emission tomography (PET) result disclosure in patients with subjective cognitive decline and explores which variables are associated with a safer disclosure in individuals who are amyloid positive.

## Introduction

Amyloid deposition in the brain is considered as the strongest risk factor for Alzheimer disease (AD),^1^ and is often also observed in patients with other neurodegenerative diseases^2^ and in cognitively unimpaired individuals.^3^ It can be assessed in vivo through positron emission tomography (PET) imaging. The use of amyloid-PET in clinical practice is recommended only for some categories of patients with objective cognitive impairment, although it is discouraged in cognitively unimpaired older individuals.^4,5^ However, consistent evidence suggests that individuals with subjective cognitive decline (SCD), a category of patients representing 21% to 29% of a general memory clinic population,^6,7^ are at higher risk of developing dementia in the upcoming years (incidence of dementia: 20.1/1000 person-years in SCD population vs 14.2/1000 person-years in controls^8^). Moreover, some additional clinical features further increase the likelihood of preclinical AD, defining the so-called subjective cognitive decline plus (SCD+) construct.^9^ Furthermore, the risk of progression to dementia is substantially increased in case of the presence of amyloid deposition (ie, a hazard ratio of 17.0 compared with SCD with normal AD biomarkers^10^). For these reasons, individuals with SCD are increasingly targeted in research and prevention initiatives,^11^ and are frequently included in research studies involving amyloid-PET (eg, ABIDE,^12^ AMYPAD-DPMS,^13,14^ DELCODE,^15^ SCIENCe,^16^ COSCODE,^17^ ALFA+^18^). Previous studies have shown that amyloid-PET has substantial clinical consequences in terms of diagnostic change and improvement of diagnostic confidence in SCD,^19,20^ and many of these individuals are proactively seeking medical advice and information about their amyloid status.^11^

Although cognitively unimpaired individuals receiving a negative amyloid-PET result are relieved as they can reinterpret their subjective memory impairment as normal aging, individuals who are amyloid-positive perceive the result as more serious and sensitive than other examinations given its implications for identity, self-determination, and stigma.^21^ Moreover, individuals without cognitive impairment who are amyloid-positive reported to contemplate and make more changes to health behaviors and future plans after disclosure than individuals who are amyloid negative.^21,22^ Nevertheless, it has been reported that the prognostic uncertainty of amyloid-PET is correctly understood by two-thirds of patients.^23^ The psychological outcomes of the amyloid-PET result disclosure in cognitively unimpaired individuals has been assessed by some previous studies. For example, one of these studies showed no association with depression and minor increases in disclosure-related distress and anxiety that are mild and well-tolerated over 6 months.^24^ Another study found that individuals who were cognitively unimpaired and receiving a positive amyloid-PET result disclosure were no more likely to experience short-term increases in anxiety, depression, or suicidality than those receiving a negative amyloid-PET result disclosure.^25^ Taken together, these results suggest that, in a research setting, the disclosure of the amyloid status in cognitively unimpaired individuals is associated with a low risk of psychological harm.^22,24,25,26,27,28^ As these studies mostly took place in research settings, evidence on the impact of the amyloid-PET result disclosure in clinical settings is currently missing. Moreover, to the best of our knowledge, no studies have empirically assessed which variables may facilitate a safer disclosure of a positive amyloid-PET result. The aims of this study were to assess (1) the psychological outcomes of the amyloid-PET result disclosure in a memory clinic population of individuals with SCD+ and (2) which variables may facilitate a safer disclosure in individuals with amyloid-positive SCD+.

## Methods

### Study Design

This study was carried out in the context of AMYPAD-DPMS. AMYPAD-DPMS is a European, multicenter, prospective, and randomized clinical study implementing amyloid-PET in clinical practice.^13^ A total of 840 memory clinic patients with variable disease stage (ie, 244 SCD+, 341 mild cognitive impairment, and 255 dementia) were recruited in 8 European memory clinics from April 16, 2018 to October 30, 2020,^14^ and allocated into 3 study groups: (1) ARM1, amyloid-PET was performed early in the diagnostic workup (ie, within 1 month from baseline); (2) ARM2, amyloid-PET was performed late in the diagnostic workup (ie, after 8 months from baseline); and (3) ARM3, amyloid-PET was performed if and when the managing physician chooses to request it (free-choice group). According to the study design, the AMYPAD-DPMS participants underwent up to 5 clinical visits: screening (baseline), 3-month, 6-month, 13-month, and 18-month (only for group 1, not mandatory) visits.^13,14^ In the present observational study, we assessed the study outcomes irrespective of participants’ randomization into the study groups (assuming that the disclosure of the amyloid-PET result has a similar outcome at different moments [eg, early or late] in the diagnostic workup, and so that randomization does not influence the outcomes of the present study).

### Participants

In the present study, participants were memory clinic patients with SCD+ who underwent amyloid-PET as part of AMYPAD-DPMS^13,14^ and who were asked to take part in an add-on study on the disclosure of the amyloid-PET result. These participants were enrolled from the 5 AMYPAD-DPMS recruiting memory clinics that accepted to participate in this study: (1) University and University Hospital of Geneva (UNIGE; Geneva, Switzerland), (2) Amsterdam University Medical Center, location VUmc (Amsterdam UMC; Amsterdam, The Netherlands), (3) Barcelonaßeta Brain Research Center (BBRC; Barcelona, Spain), (4) University College London (UCL; London, United Kingdom), and (5) Centre Hospitalier Universitaire Vaudois (CHUV; Lausanne, Switzerland). Criteria to define SCD+ were based on a modified version of the SCD-I Working group criteria,^9^ of which the most relevant features are age between 60 and 85 years, perceived decline in memory over time, SCD onset within the previous 5 years and duration greater than 6 months, Mini-Mental State Examination (MMSE) score between 27 to 30, exclusion of MCI, explicit concerns (worries) about the cognitive symptoms, and active seeking of consultation. More information on inclusion and exclusion criteria was reported in previous papers.^13,14^

The Ethics Committees of all recruiting memory clinics approved the study. All participants gave written informed consent. The trial was registered (2017-002527-21). This report follows the Strengthening the Reporting of Observational Studies in Epidemiology (STROBE) reporting guideline for observational studies.

### Disclosure Modalities

In AMYPAD-DPMS, amyloid-PET scans were acquired with either [18F]flutemetamol (Vizamyl, GE Healthcare, Amersham United Kingdom) or [18F]florbetaben (Neuraceq, Life Molecular Imaging, Berlin, Germany) as amyloid-PET tracers, and visually assessed by trained local nuclear medicine physicians using reading methods approved by the US Food and Drug Administration and the European Medicines Agency for the 2 tracers. The AMYPAD consortium recommended the disclosure of the amyloid-PET result in participants with SCD+. Before the beginning of participants’ recruitment, we developed ad hoc nonprescriptive disclosure guidelines (eTable 1 in Supplement 1) adapted from those used in the A4 trial^29^ and a brochure template (eTable 2 in Supplement 1) to support managing physicians in the disclosure process. Nevertheless, managing physicians may or may not have followed the disclosure guidelines, and delivered, all or in part, information outlined in the disclosure brochure template. Moreover, we recorded whether it was communicated that SCD+ is a risk condition for the future development of cognitive impairment or dementia, whether the amyloid-PET result was disclosed in person (face-to-face) or over the phone, and the disclosure duration by using an ad hoc questionnaire.

### Outcomes

In the present study we assessed: (1) the psychological outcomes after the amyloid-PET result disclosure in a memory clinic population of SCD+ individuals, and (2) which variables were associated with a safer disclosure in patients with amyloid-positive SCD+. The psychological outcomes after the amyloid-PET result disclosure were defined as disclosure-related distress, assessed using the Impact of Event Scale–Revised (IES-R); and anxiety and depression, assessed using the Hospital Anxiety and Depression scale (HADS).

IES-R a 22-item self-report measure assessing subjective distress caused by traumatic events (ie, the amyloid-PET result disclosure in this case) that occurred in the past 7 days, and consists of 1 total score (0 to 88) and 3 average subscores (0 to 4): avoidance (avoidance of thoughts, feelings, memories or situations), intrusions (intrusive memories, thoughts, or feelings causing distress), and hyperarousal (hypervigilance, feeling watchful and on guard, difficulty concentrating), with higher scores meaning more symptoms. The IES-R total score is normally used to support a clinical diagnosis of posttraumatic stress disorder (PTSD; greater than or equal to 33: probable presence of a PTSD).^30^ In previous studies, the IES-R total score has been used to assess the presence of PTSD-like symptoms in the context of medical bad news delivery (eg, cancer diagnosis^31^), and also to assess the disclosure-related distress in patients undergoing amyloid-PET.^22,27,28^ The IES-R was administered to all participants included in this study 1 to 3 days after the amyloid-PET result disclosure.

HADS is a 14-item self-report screening scale containing two 7-item scales: one for anxiety and one for depression, both with a score range of 0 to 21, with higher scores meaning higher levels of anxiety or depression.^32^ Items referring to symptoms that may have a physical cause (eg, insomnia and weight loss) are not included in the scale, so the HADS is considered to be unbiased by coexisting general medical conditions.^33^ HADS has 2 distinct subscales (HADS Anxiety and HADS Depression) and scores of at least 15 indicate the probable presence of severe mood disorder symptoms, and scores of at least 8 indicate mild symptoms.^34^ HADS was administered to all participants at different times during the study. For the present study, we used HADS scores collected at baseline and after the amyloid-PET result disclosure (the time interval between the baseline and the follow-up HADS administrations is not consistent among participants). Deltas (follow-up HADS score minus the baseline HADS score) were used to assess changes in anxiety and depression after amyloid-PET. For participants who underwent amyloid-PET before the 3-month visit, we also assessed anxiety and depression after 13 months (ie, the last visit in which HADS was administered).

In order to assess which variables were associated with a safer amyloid-PET result disclosure in individuals with amyloid-positive SCD+, we assessed the association of age, sex, education (years), global cognition (MMSE score), communication that SCD+ is a risk condition, disclosure type (face-to-face vs phone), disclosure duration, and presence of study partner (independent variables) with IES-R total and subscale scores, and HADS Anxiety and Depression (dependent variables).

### Statistical Analysis

Continuous variables are described as median and IQR, and categorical variables as percentages (raw numbers). Between-group differences were assessed using Wilcoxon rank sum test for continuous variables, or test for equality of proportions for categorical variables. Significance was set at 2-tailed *P* < .05, and post hoc pairwise comparisons (Dunn all-pairs rank comparison test for continuous variables, or pairwise comparisons for proportions) were adjusted using Bonferroni correction. Associations between continuous variables were assessed using Spearman correlations (ρ). All statistical analyses were performed with R, version 4.1.2 (R Project for Statistical Computing) from July to October 2022.

## Results

### Participants

A total of 105 participants with SCD+ of AMYPAD-DPMS, who received their amyloid-PET results, participated in this study (see eFigure 1 in Supplement 1 for the study flowchart and eFigure 2 in Supplement 1 for geographical distribution). Among them, 26% (27 of 105) were assessed as amyloid positive and 74% (78 of 105) as amyloid negative. Table 1 illustrates their main sociodemographic and clinical features in the whole group and disaggregating by amyloid status. No significant differences in the main sociodemographic and clinical features were found between participants with amyloid-positive SCD+ and amyloid-negative SCD+ (median [IQR] age: 70 [66 to 74] years vs 67 [64 to 74] years; *W* = 1269.5; *P* = .11; gender: 52% men [14 of 27] vs 58% men [45 of 78]; χ^2^ = 0.09, *P* = .76; median [IQR] years of education: 15 [13 to 17] years vs 15 [12 to 17] years; *W* = 1076.5; *P* = .87; median [IQR] MMSE score: 29 [28 to 30] vs 29 [28 to 30]; *W* = 1090.0; *P* = .78).

**Table 1.** Features of the Participants With Subjective Cognitive Decline Plus in the Amyloid Imaging to Prevent Alzheimer Disease Diagnostic and Patient Management Study and Disclosure Modalities in the Whole Group and Disaggregating by Amyloid Status

Features	Whole group, No. (%) (N = 105)	By amyloid status		
		Positive, No. (%) (n = 27)	Negative, No. (%) (n = 78)	*P* value
Age, median (IQR), y	69 (64-74)	70 (66-74)	67 (64-74)	.11
Gender				
Men	59 (56)	14 (52)	45 (58)	.76
Women	46 (44)	13 (48)	33 (42)	
Education, median (IQR), y	15 (12-17)	15 (13-17)	15 (12-17)	.87
MMSE, median (IQR)	29 (28-30)	29 (28-30)	29 (28-30)	.78
Communication that SCD+ is a risk condition for the future development of cognitive impairment and dementia (yes vs no)	99 (94)	26 (96)	73 (94)	.97
Face-to-face disclosure	67 (64)	22 (81)	45 (58)	.047
Disclosure duration, median (IQR), min	30 (25-60)	52 (30-60)	30 (20-35)	<.001
Presence of study partner	7 (7)	3 (11)	4 (5)	.53

Abbreviations: MMSE, Mini Mental State Examination; SCD+, subjective cognitive decline plus.

### Disclosure Modalities

Table 1 illustrates the disclosure modalities in the whole group and by amyloid status. In 94% of participants with SCD+ (99 of 105), it was communicated that SCD+ is a risk condition for the future development of cognitive impairment or dementia. Among those to whom it was not communicated that SCD+ is a risk condition (n = 6), reasons for not communicating were available only for 5 participants (who were all amyloid negative): amyloid-PET was negative (n = 3), amyloid-PET was negative and the medial temporal lobe atrophy scale score was 0 (n = 1), there was a clear psychosocial factor for the SCD (n = 1).

In participants who were amyloid positive, disclosure was more frequently done face to face rather than over the phone (81% [22 of 27] vs 58% [45 of 78]; χ^2^ = 3.9, *P* = .047) and lasted longer (median [IQR] time: 52 [30 to 60] vs 30 [20 to 35] minutes; *W* = 1486.5, *P* < .001) than in participants who were amyloid negative. It is important to note that at a certain point (approximatively corresponding to March 2020) the number of disclosures done over the phone increased due to the COVID-19 pandemic (7% [4 of 56] before and 69% [34 of 49] after March 1, 2020). Disclosure was done face to face in 87% (13 of 15) of participants who were amyloid-positive SCD+ and 95% (39 of 41) of participants who were amyloid negative (χ^2^ = 0.3; *P* = .62) before March, 2020; and in 75% (9 of 12) of amyloid-positive participants and 16% (6 of 37) of amyloid-negative participants (χ^2^ = 12.1; *P* < .001) after March 1, 2020.

### Association of Amyloid-PET Disclosure and Distress, Anxiety, and Depression

**Figure 1.**
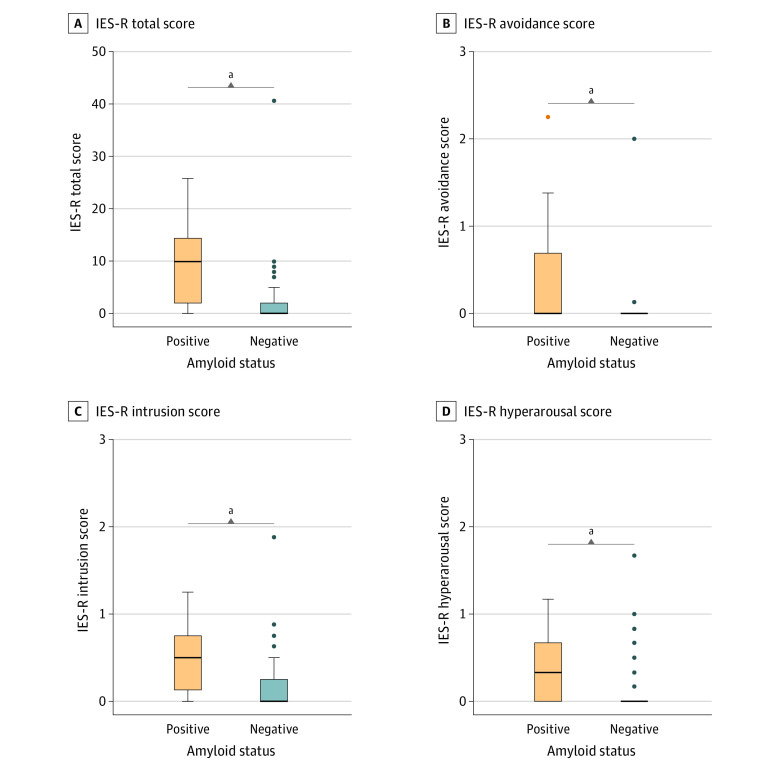
Association of the Amyloid–Positron Emission Tomography Disclosure With Disclosure-Related Distress in Individuals With Subjective Cognitive Decline Plus Who Had Amyloid-Positive and Amyloid-Negative Results Impact of Events Scale–Revised (IES-R) total score (range, 0-88; at least 33 indicates probable presence of a posttraumatic stress disorder). Submeasures include IES-R Avoidance Score, Intrusion Score, and Hyperarousal Score (range, 0-4, no thresholds available). IES-R was administered 1 to 3 days after the amyloid–positron emission tomography result disclosure. The horizontal line inside the box indicates the median; lower and upper hinges of the box, 25th and 75th percentiles, respectively; lower whisker, smallest observation greater than or equal to lower hinge − 1.5 × IQR; upper whisker, largest observation less than or equal to upper hinge + 1.5 × IQR; and individual points (when present), outliers. ^a^Indicates statistically significant difference (*P* < .05).

**Figure 2.**
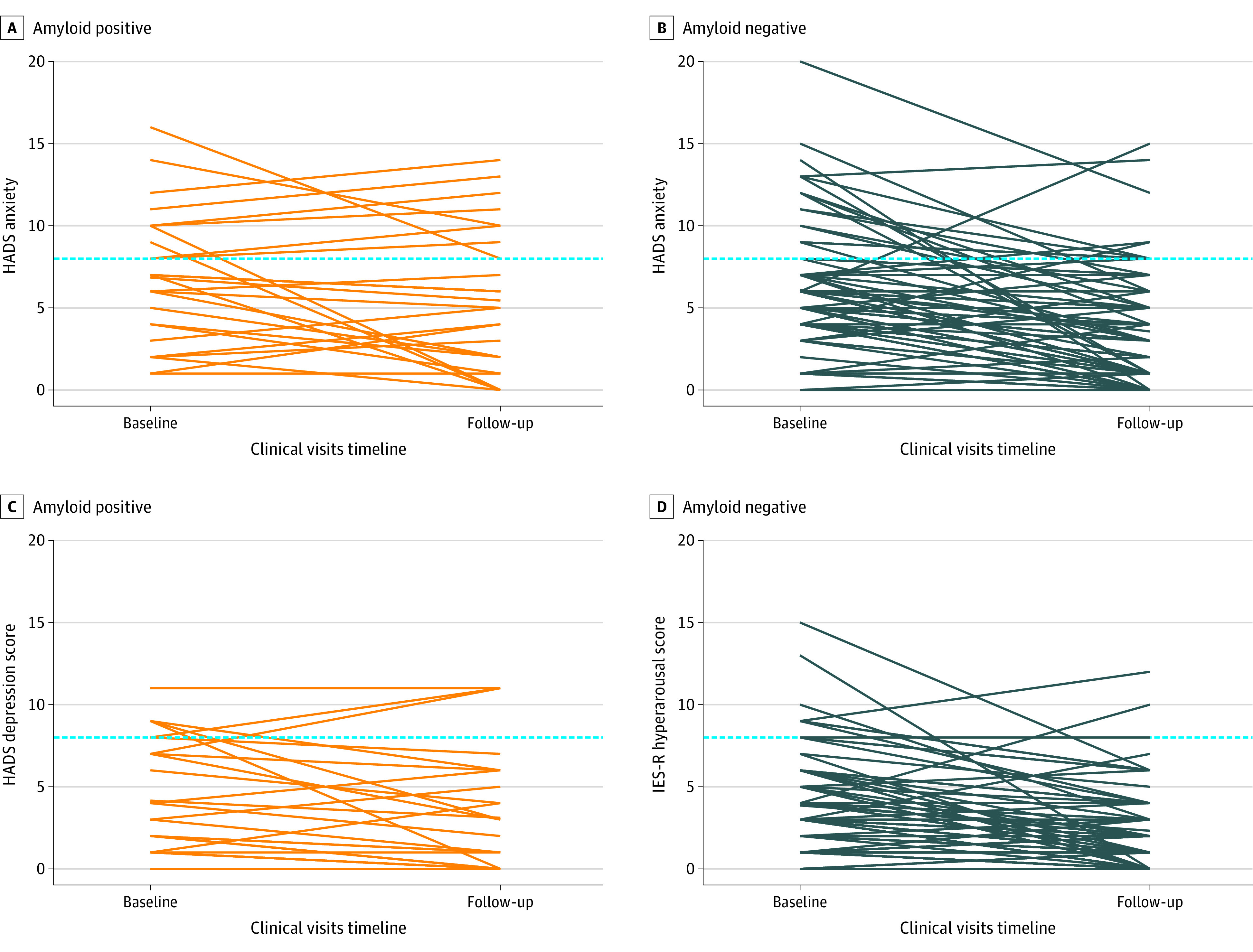
Association of the Amyloid–Positron Emission Tomography Disclosure With Anxiety and Depression in Individuals With Subjective Cognitive Decline Plus Who Had Amyloid-Positive or Amyloid-Negative Results Hospital Anxiety and Depression scale (HADS) Anxiety and Depression scores (range 0-21, at least 15 indicates severe anxiety and/or depression symptoms). HADS was administered at baseline and after the amyloid-PET result disclosure. Dashed horizontal lines represent the threshold defining at least mild (HADS score ≥8) anxiety and/or depression symptoms. The prevalence of at least mild anxiety symptoms at follow-up was 30% (8 of 27 individuals) in individuals with amyloid-positive PET findings and 13% (10 of 78 individuals; χ^2^ = 2.9, *P* = .09) in individuals with amyloid-negative PET findings. The prevalence of at least mild depression symptoms at follow-up was 11% (3 of 27 individuals) in individuals with amyloid-positive PET findings and 4% (3 of 78 individuals; χ^2^ = 0.8, *P* = .36) in individuals with amyloid-negative PET findings.

After disclosure, patients with amyloid-positive SCD+ had significantly higher median (IQR) IES-R total score (10 [2 to 14] vs 0 [0 to 2]; *W* = 1721.0; *P* < .001), IES-R avoidance (0.00 [0.00 to 0.69] vs 0.00 [0.00 to 0.00]; *W* = 1520.0; *P* < .001), IES-R intrusions (0.50 [0.13 to 0.75] vs 0.00 [0.00 to 0.25]; *W* = 1644.5; *P* < .001), and IES-R hyperarousal (0.33 [0.00 to 0.67] vs 0.00 [0.00 to 0.00]; *W* = 1562.0, *P* < .001) compared with patients who were amyloid negative (Figure 1). We observed no differences between participants with amyloid-positive SCD+ and participants with amyloid-negative SCD+ in the median (IQR) HADS Anxiety (–1.0 [–3.0 to 1.8] vs –2.0 [–4.8 to –1.0]; *W* = 1267.5; *P* = .06) and median (IQR) Depression (–1.0 [–2.0 to 0.0] vs –1.0 [–3.0 to 0.0]; *W* = 1112.0; *P* = .46) deltas (scores after disclosure minus scores at baseline) (Figure 2). A total of 63 participants with SCD+ underwent amyloid-PET before the 3-month follow-up visit, and 46 (15 amyloid positive and 31 amyloid negative) repeated HADS after 13 months. Among them, participants who were amyloid positive and participants who were amyloid negative showed similar median (IQR) HADS Anxiety (–1.0 [–1.0 to 0.5] vs –1.0 [–2.0 to 0.0]; *W* = 252.0; *P* = .65) and median (IQR) Depression (0.0 [–2.0 to 1.5] vs –1.0 [–2.0 to 0.0]; *W* = 264.5; *P* = .45) deltas (scores after 13 months minus scores at baseline).

No participants with amyloid-positive SCD+ showed probable presence of PTSD (ie, IES-R total score at least 33) (Figure 1), or severe anxiety or depression symptoms (ie, HADS score at least 15) at follow-up (Figure 2). Moreover, amyloid-positive and amyloid-negative SCD+ showed similar prevalence of at least mild (ie, HADS score at least 8) anxiety (30% [8 of 27] vs 13% [10 of 78]; χ^2^ = 2.9; *P* = .09) or depression (11% [3 of 27] vs 4% [3 of 78]; χ^2^ = 0.8; *P* = .36) symptoms at follow-up (Figure 2).

### Variables Facilitating a Safer Disclosure in Amyloid-Positive Individuals

**Figure 3.**
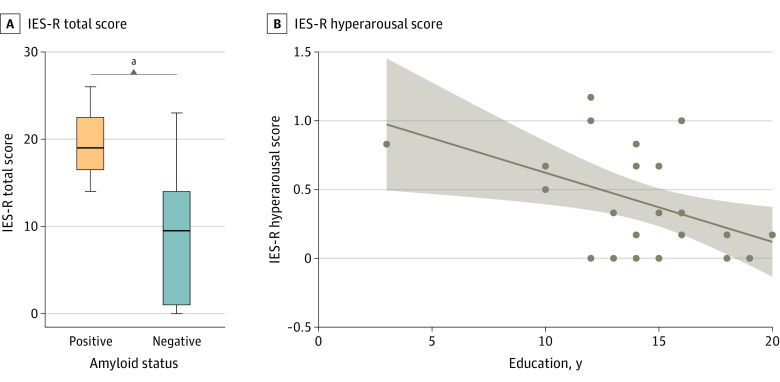
Variables Associated With a Safer Disclosure in Individuals With Subjective Cognitive Decline Plus and Amyloid-Positive Results A, The horizontal line inside the box indicates the median; lower and upper hinges of the box, 25th and 75th percentiles, respectively; lower whisker, smallest observation greater than or equal to lower hinge − 1.5 × IQR; upper whisker, largest observation less than or equal to upper hinge + 1.5 × IQR; and individual points (when present), outliers. B, line indicates regression estimate; shading, 95% CI; dots, individual data points. ^a^Indicates statistically significant difference (*P* < .05).

In participants with amyloid-positive SCD+ (n = 27), the presence of study partner was associated with higher IES-R total score (*W* = 7.5; *P* = .03). Higher education was associated with lower IES-R hyperarousal (ρ = –0.43, *P* = .02) (Table 2 and Figure 3).

**Table 2.** Variables Facilitating a Safer Disclosure in Individuals With SCD+ and Amyloid-Positive Results

Features	IES-R								Changes in HADS			
	Total		Avoidance		Intrusions		Hyperarousal		Anxiety		Depression	
	Index, *W*	*P* value	Index, *W*	*P* value	Index, *W*	*P* value	Index, *W*	*P* value	Index, *W*	*P* value	Index, *W*	*P* value
Age (y), ρ	–0.15	.45	–0.16	.44	–0.19	.35	0.07	.73	–0.02	.91	–0.25	.23
Gender	92.0	.98	91.5	1.00	102.0	.61	70.5	.32	85.5	.98	106.5	.26
Education (years), ρ	–0.18	.37	–0.13	.51	–0.07	.73	–0.43	.02	–0.15	.48	–0.15	.46
MMSE, ρ	0.21	.29	0.35	.07	–0.01	.95	0.04	.83	0.23	.26	–0.10	.61
Communication that SCD+ is a risk condition	1.5	.16	6.5	.41	2.0	.17	3.5	.24	14.0	.89	18.0	.50
Disclosure type (face to face vs phone)	72.0	.30	64.5	.54	68.5	.41	65.0	.55	51.0	.64	27.0	.23
Disclosure duration (min), ρ	0.02	.93	0.16	.44	0.03	.90	–0.31	.13	–0.28	.18	–0.27	.19
Presence of study partner	7.5	.03	21.0	.23	11.5	.06	15.0	.11	49.0	.26	24.0	.41

Abbreviations: HADS, Hospital Anxiety and Depression scale; IES-R, Impact of Events Scale–Revised; MMSE, Mini Mental State Examination; SCD+, subjective cognitive decline plus.

## Discussion

Our results found that the disclosure of a positive amyloid-PET result to individuals with SCD+ was associated with a greater change in psychological well-being than a negative amyloid-PET result, but this change did not reach the threshold for clinical concern. Specifically, patients with amyloid-positive SCD+ experienced higher levels of avoidance, intrusion, and hyperarousal than amyloid-negative patients directly after disclosure. In contrast, the disclosure of the amyloid-PET result had no statistically significant differences for anxiety and depression in individuals with amyloid-positive and amyloid-negative SCD+ (also in a subgroup of participants with a longer follow-up at 13 months). Finally, in individuals with SCD+ receiving a positive amyloid-PET result, we observed that higher education was associated with lower hyperarousal symptoms (hypervigilance, feeling watchful and on guard, difficulty concentrating), and that disclosure-related distress was higher when a study partner was present.

Our results are in line with previous studies demonstrating that the disclosure of a positive amyloid-PET result does not cause significant psychological risk. Moreover, to the best of our knowledge, this is the first study exploring which variables may facilitate a safer disclosure of a positive amyloid-PET result. In particular, higher education might help participants elaborate and interpret the meaning of a positive biomarker result, and cope with its psychological consequences. Thus, special attention should be paid to individuals with lower education levels during the disclosure. In addition, although the number of participants with SCD+ who were accompanied by a study partner was very small, we can speculate that these individuals were more sensitive to the disclosure of positive biomarker results or might have been more distressed by the worry of how this information may change their families’ and friends’ perceptions of them than participants with SCD+ without study partners. Concerning risk communication, a recent study found that the use of some communication strategies might maximize the information uptake in mild cognitive impairment patients, thus resulting in higher trust and satisfaction.^35^

Delivering bad news to cognitively unimpaired patients could be one of the most challenging tasks physicians have to face in their clinical practice. Therefore, it is crucial to highlight that disclosing a positive amyloid-PET result was not associated with a clinically relevant psychological change. Comparing the psychological changes associated with the disclosure of a positive amyloid-PET result with the psychological changes associated with a cancer diagnosis, we qualitatively observed higher distress, anxiety, and depression levels in oncological patients,^31^ consistently with the nature and meaning of the disclosed result (cancer diagnosis: presence of a disease that might reduce life expectancy; amyloid-PET positivity: high risk of a disease that might reduce life expectancy).

The AMYPAD consortium recommended the disclosure of the amyloid-PET result in participants with SCD+. One could argue that, as AMYPAD-DPMS is a research project, this propensity to disclose (physicians’ perspective) and receive (patients’ perspective) the amyloid-PET result might be different in a clinical setting. However, our sample is representative of a wider memory clinic population,^14^ and we believe that our results are generalizable to the clinical practice of memory clinics where individuals who are cognitively unimpaired proactively seek medical help and therefore expect to undergo medical examination and receive their results. This is supported by previous studies reporting that cognitively unimpaired individuals support the amyloid-PET result disclosure,^26,36,37^ and by a survey showing that patients and caregivers placed greater value on testing in asymptomatic individuals than clinicians.^38^

### Limitations

The main limitations of the present study are the lack of long-term assessment of health-related outcomes (eg, quality of life) and the lack of follow-up on the psychological outcomes of amyloid-PET disclosure. Indeed, the absence of follow-up data prevents us from alleging that the disclosure of a positive amyloid-PET does not cause significant psychological risk in the long term. Another issue is the relatively small sample size, possibly resulting in insufficient power to detect small associations and preventing us from drawing strong conclusions. Moreover, only some of the multiple aspects of psychological risk have been assessed (ie, distress, depression, and anxiety), possibly neglecting relevant issues such as suicidal ideation, which is a potential negative outcome reported in previous studies of risk disclosure.

## Conclusions

This study found that the disclosure of a positive amyloid-PET result to participants with SCD+ was associated with greater psychological changes, yet such changes did not reach the threshold for clinical concern. This study adds evidence to previous studies showing that the disclosure of the amyloid-PET result is associated with a low risk of psychological harm.

## Notes

Supplement 1. **eTable 1.** Non-Prescriptive Disclosure Guidelines for the Disclosure of Amyloid-PET Result to SCD+ Participants in AMYPAD-DPMS (Adapted From: Harkins et al., 2015) **eTable 2.** Non-Prescriptive Brochure Template for the Disclosure of the Amyloid-PET Result to SCD+ Participants in AMYPAD-DPMS **eFigure 1.** Study Flowchart **eFigure 2.** Geographical Distribution of the SCD+ Participants
eReference

Supplement 2.
Nonauthor Collaborators

Supplement 3.
Data Sharing Statement
