# Error in Supplement 2

**DOI:** 10.1001/jamanetworkopen.2023.5066

**Published:** 2023-03-08

In the Original Investigation titled “Analysis of Psychological Symptoms Following Disclosure of Amyloid–Positron Emission Tomography Imaging Results to Adults With Subjective Cognitive Decline,”^1^ published January 13, 2023, a nonauthor collaborator’s name was misspelled in Supplement 2. This article has been corrected.^1^
