# Supplementary material for: Analysis of Psychological Symptoms Following Disclosure of Amyloid–Positron Emission Tomography Imaging Results to Adults With Subjective Cognitive Decline
Source: JAMA Netw Open. 2023 Jan 13;6(1):e2250921. doi: 10.1001/jamanetworkopen.2022.50921 (PMC9857261; doi:10.1001/jamanetworkopen.2022.50921)
Supplement: Supplement 1. — eTable 1. Non-Prescriptive Disclosure Guidelines for the Disclosure of Amyloid-PET Result to SCD+ Participants in AMYPAD-DPMS (Adapted From: Harkins et al., 2015) eTable 2. Non-Prescriptive Brochure Template for the Disclosure of the Amyloid-PET Result to SCD+ Participants in AMYPAD-DPMS eFigure 1. Study Flowchart eFigure 2. Geographical Distribution of the SCD+ Participants eReference [file jamanetwopen-e2250921-s001.pdf]

Supplemental Online Content

Caprioglio C, Ribaldi F, Visser LNC, et al; AMYPAD consortium. Analysis of psychological symptoms following disclosure of amyloid–positron emission tomography imaging results to adults with subjective cognitive decline. *JAMA Netw Open.* 2023;6(1):e2250921. doi:10.1001/jamanetworkopen.2022.50921

eTable 1. Non-Prescriptive Disclosure Guidelines for the Disclosure of Amyloid-PET Result to SCD+ Participants in AMYPAD-DPMS (Adapted From: Harkins et al., 2015)

eTable 2. Non-Prescriptive Brochure Template for the Disclosure of the Amyloid-PET Result to SCD+ Participants in AMYPAD-DPMS

eFigure 1. Study Flowchart

eFigure 2. Geographical Distribution of the SCD+ Participants

eReference

This supplemental material has been provided by the authors to give readers additional information about their work.

© 2023 Caprioglio C et al. *JAMA Network Open.*

eTable 1. Non-prescriptive disclosure guidelines for the disclosure of amyloid-PET result to SCD+ participants in AMYPAD-DPMS (adapted from: Harkins et al., 2015[1]).

**Best practice**	**Details**
Step 0: Prior to in-person screening	
Send Amyloid Imaging Disclosure Process brochure prior to consent.	Brochure content descriptions are provided in Table S2.
Step 1A: Education and informed consent	
Assess knowledge of study and role of amyloid imaging in study.	Use this information to structure review of the brochure.
Assess motivation for joining study.	Example questions:<br>Tell me what you know about an amyloid-PET scan?<br>Why are you interested in having an amyloid-PET scan?<br>Why are you interested in joining the study?
Conduct educational session.	Cover brochure contents and tailor based on participant's prior knowledge.<br>Study staff should be skilled in communication.<br>Introduce the concept of risk.<br>Explain meaning of elevated* amyloid on PET scan, clarify that this does not necessarily mean that an individual will develop symptoms of Alzheimer's disease.<br>Benchmark the risk of brain amyloid with other dementia and non-dementia risks.
Assess understanding of brochure.	Use "teach back" method: "Can you tell me in your own words what we just talked about?"<br>Focus on understanding of amyloid imaging and its role in study.
Step 1B: Screening assessments	
Screen for anxiety and depression (for example: State-Trait Anxiety Inventory (STAI), Geriatric Depression Scale (GDS), Hospital Anxiety and Depression Scale (HADS)).	Decisions on eligibility will be study-specific and involve investigator's clinical judgment.
Step 2: Amyloid-PET scan	
Participant undergoes amyloid-PET scan.	Conduct imaging on a separate day from consent.<br>Do not disclose results on the day of imaging.
Step 3A: Amyloid status disclosure - pre-disclosure	
Assess mood.<br>Assess recent life stress.	Investigator/study staff should be skilled in communication and recognition of distress.

© 2023 Caprioglio C et al. *JAMA Network Open.*

Assess willingness to receive result.	If concerns arise, discuss possibility of delaying disclosure (if the educational session has been well conducted, this should be exceptional).
Step 3B: Amyloid status disclosure	
Disclose amyloid status using language from the brochure.	Use the same concepts of the educational session.<br>Disclose in-person, with time for questions.<br>Give participant option of having family member or friend present.<br>Provide a written summary.
Assess understanding of amyloid status result.	Example questions:<br>What does that mean to you?<br>Do you have any questions about your result?
Step 4: Post-disclosure follow-up (only if a project on communication of amyloid-PET is put in place)	
Conduct follow-up phone call one to three days post-disclosure.	Assess well-being, distress, and impact of disclosure (for example: Impact of Event Scale, IES).<br>Answer questions.<br>Create appropriate follow-up plan based on participant's responses.
Step 5: Follow-up over study course (only if a project on communication of amyloid-PET is put in place)	
Assess anxiety, depression, impact of disclosure (for example: STAI, GDS, HADS, IES).	Study protocol should specify frequency of assessments and plans for additional monitoring if distress is observed.

* In AMYPAD-DPMS, amyloid-PET scans were acquired with either [18F]flutemetamol (Vizamyl, GE Healthcare, Amersham United Kingdom) or [18F]florbetaben (Neuraceq, Life Molecular Imaging, Berlin, Germany) as amyloid-PET tracers, and visually assessed by trained local nuclear medicine physicians using FDA/EMA-approved reading methods for the two tracers.

© 2023 Caprioglio C et al. *JAMA Network Open.*

eTable 2. Non-prescriptive brochure template for the disclosure of the amyloid-PET result to SCD+ participants in AMYPAD-DPMS.

Brochure topic	Key points
What is the AMYPAD-DPMS trial?	• The AMYPAD Diagnostic and Patient Management Study (AMYPAD-DPMS) is a trial aiming to test the impact of amyloid-PET on the diagnosis and treatment that your physician will give you.
Why is amyloid-PET being tested?	• Amyloid-PET is known to safely provide reliable information about the presence or absence of amyloid in your brain.<br>• However, we do not know whether adding this exam to those that would be normally prescribed to you provides any advantage to your health.<br>• As amyloid-PET is an expensive procedure, this is a relevant question to answer.
What will happen if I enroll in the AMYPAD-DPMS trial?	You will be randomly assigned to one of the following three options:<br>(i) you will undergo amyloid-PET right after MR scan. After knowing the result of the exam, your physician will be free to choose whatever other exam he/she feels fit;<br>(ii) you will undergo the diagnostic exams that your physician would use regardless of the AMYPAD-DPMS trial to establish a diagnosis and give you treatment. You will undergo amyloid-PET in 6 to 10 months' time, after which your physician may or may not change his/her initial diagnosis and treatment;<br>(iii)you will undergo amyloid-PET if your physician so decides, and whenever he/she feels fit.
What is Alzheimer's disease?	• Alzheimer's disease is a brain disease.<br>• It is the most common cause of dementia.<br>• Common symptoms of dementia caused by Alzheimer's disease are problems with memory and thinking that impair a person's ability to do their usual and everyday activities.<br>• As persons with Alzheimer's disease develop symptoms, the first to appear are memory and thinking problems that are bothersome but do not interfere

© 2023 Caprioglio C et al. *JAMA Network Open.*

	with daily activities. Over time, usually several years, as these problems worsen, the person develops dementia.
What is amyloid?	• Amyloid is a protein in the brain.<br>• In Alzheimer's disease, amyloid builds up and brain function gets worse<br>• Amyloid can sometimes be detected years before a person has noticeable memory problems.
How do we know whether someone has brain amyloid?	• An amyloid-PET scan measures brain amyloid.
What does having a brain amyloid scan involve?	• An injection of a radioactive drug.<br>• The scan measures the level of amyloid in your brain.
What does an elevated* level of brain amyloid mean?	An 'elevated* amyloid' result:<br>• means that amyloid plaques are present in your brain;<br>• does not mean you now have Alzheimer's disease dementia or that you will certainly get Alzheimer's disease dementia;<br>• means that you are at greater risk to get Alzheimer's disease dementia in the coming years than someone who has a 'not elevated*' amyloid result;<br>• means you may be eligible to join Alzheimer's prevention trials that will test anti-amyloid therapies.
Is an elevated* level of amyloid like other medical risks?	• The relationship between elevated* amyloid and Alzheimer's disease dementia is similar to the relationship between high cholesterol and heart disease.<br>• Many factors may protect a person from developing memory or thinking problems even if they have elevated* levels of amyloid.<br>• Good general health and a healthy lifestyle are known to lower the risk of Alzheimer's disease dementia.
What does a not elevated* level of	• A 'not elevated*' amyloid result means that it is unlikely you have amyloid plaques in your brain at this time.

© 2023 Caprioglio C et al. *JAMA Network Open.*

brain amyloid mean?	• A person who has a 'not elevated*' amyloid level could develop an 'elevated*' level and Alzheimer's disease dementia in the future, but not before a sizable number of years.

* In AMYPAD-DPMS, amyloid-PET scans were acquired with either [18F]flutemetamol (Vizamyl, GE Healthcare, Amersham United Kingdom) or [18F]florbetaben (Neuraceq, Life Molecular Imaging, Berlin, Germany) as amyloid-PET tracers, and visually assessed by trained local nuclear medicine physicians using FDA/EMA-approved reading methods for the two tracers.

© 2023 Caprioglio C et al. *JAMA Network Open.*

eFigure 1. Study flowchart.

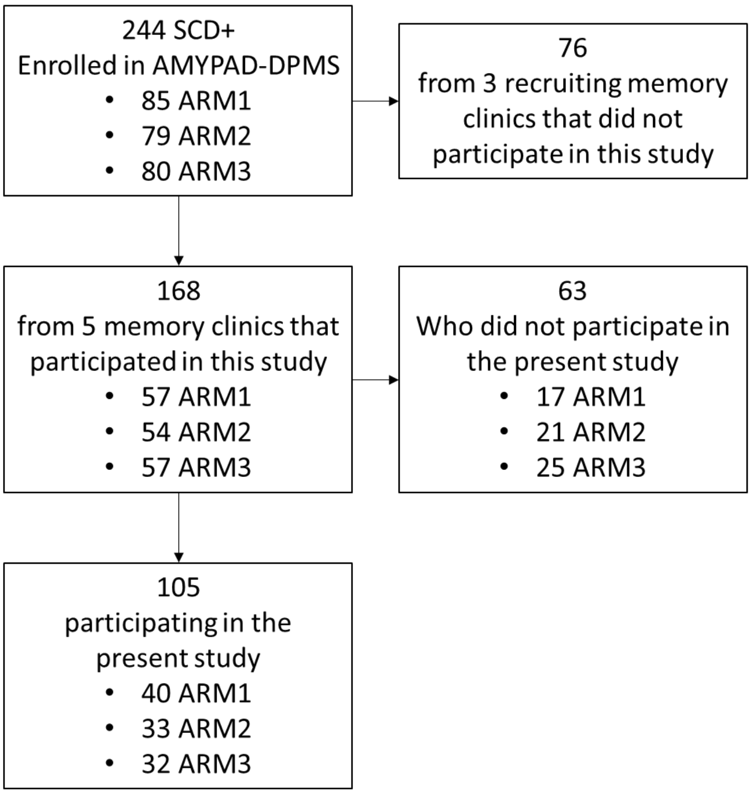

The present study was started after the beginning of AMYPAD-DPMS recruitment. Therefore, not all AMYPAD-DPMS SCD+ participants were invited to participate.

© 2023 Caprioglio C et al. *JAMA Network Open.*

eFigure 2. Geographical distribution of the SCD+ participants.

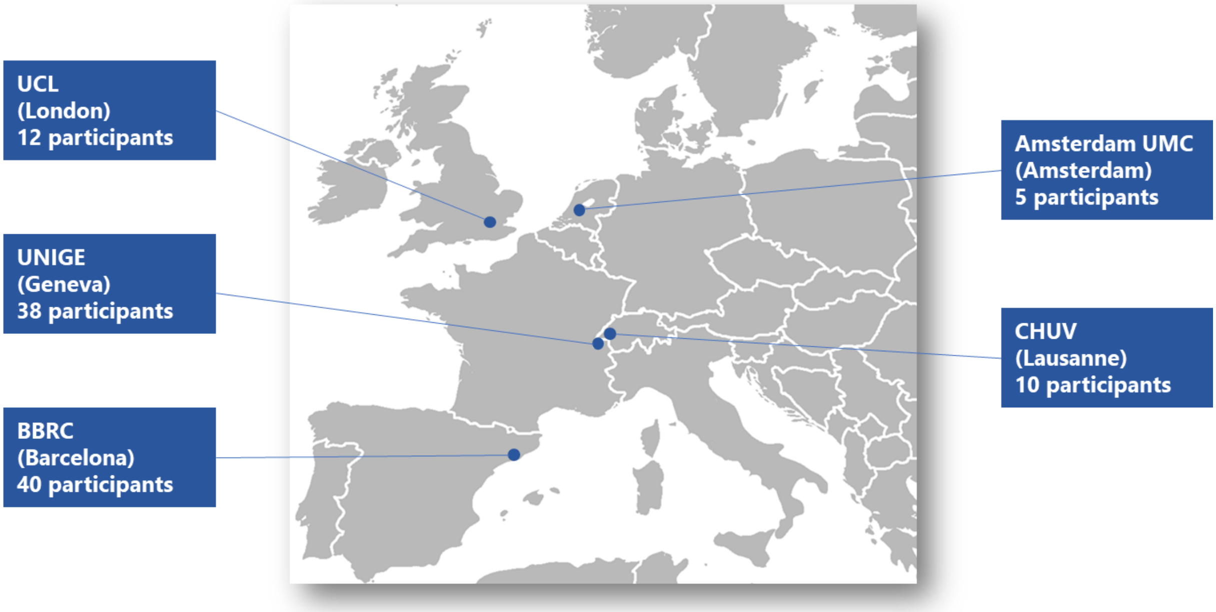

UNIGE: University and University Hospital of Geneva. Amsterdam UMC: Amsterdam University Medical Centers, location VUmc. BBRC: Barcelonaβeta Brain Research Center. UCL: University College London. CHUV: Centre Hospitalier Universitaire Vaudois.

© 2023 Caprioglio C et al. *JAMA Network Open.*

eReference

1 Harkins K, Sankar P, Sperling R, *et al.* Development of a process to disclose amyloid imaging results to cognitively normal older adult research participants. *Alzheimer's Res Ther* 2015; **7**. DOI:10.1186/s13195-015-0112-7.

© 2023 Caprioglio C et al. *JAMA Network Open.*
