# Supplementary material for: Analysis of Psychological Symptoms Following Disclosure of Amyloid–Positron Emission Tomography Imaging Results to Adults With Subjective Cognitive Decline
Source: JAMA Netw Open. 2023 Jan 13;6(1):e2250921. doi: 10.1001/jamanetworkopen.2022.50921 (PMC9857261; doi:10.1001/jamanetworkopen.2022.50921)
Supplement: Supplement 2. — Nonauthor Collaborators [file jamanetwopen-e2250921-s002.pdf]

Supplemental Online Content: Nonauthor Collaborators
*First name, last name, and suffix (if applicable) are required and will appear in PubMed.

*Group Name(s): AMYPAD consortium

*First Name and Middle Initial(s)	*Last Name	*Suffix (eg, Jr, III)	Academic Degrees	Institution	Location (city, state/province, country)	Role or Contribution, eg, chair, principal investigator	Group (if more than 1 Group listed in the byline) and/or Subgroup (eg, Steering Committee)
Carla	Abdelnour						
Nuria	Aguilera						
Leon	Aksman						
Emilio	Alarcón-Martín						
Montse	Alegret						
Silvia	Alonso-Lana						
Daniele	Altomare						
Pia	Andersen						
Majd	Arab						
Malin	Aspö						
Ilona	Bader						
Ilse	Bader						
Nigel	Banton						
Frederik	Barkhof						
Rodrigo	Barnes						
Dawn	Barrie						
Mark	Battle						
Ana	Belén Collado						
Julie	Bellet						
Johannes	Berkhof						
Marine	Biger						
Cindy	Birck						
Gerard	Bischof						
Mercè	Boada						
Ronald	Boellaard						
Nenad	Bogdanovic						
Ariane	Bollack						
Stéphanie	Bombois						
Stefan	Borg						

*First name, last name, and suffix (if applicable) are required and will appear in PubMed.

***First Name and Middle Initial(s)**	***Last Name**	***Suffix (eg, Jr, III)**	Academic Degrees	Institution	Location (city, state/province, country)	Role or Contribution, eg, chair, principal investigator	Group (if more than 1 Group listed in the byline) and/or Subgroup (eg, Steering Committee)
Anne	Borjesson-Hanson						
Vladimir	Boskov						
Justine	Boutantin						
Claire	Boutoleau-Bretonniere						
Femke	Bouwman						
Laetitia	Breuilh						
Eva	Bringman						
Baptiste	Brunel						
Marco	Bucci						
Chris	Buckley						
Mar	Buendía						
Santi	Bullich						
Anna	Calvet						
Laia	Cañada						
Marta	Cañada						
Camilla	Caprioglio						
Jorge	Cardoso						
Jasmine	Carlier						
Elise	Carre						
Isabelle	Carrie						
Pascaline	Cassagnaud						
Emmanuelle	Cassol						
Miguel	Castilla-Martí						
Elodie	Cazalon						
Tiphaine	Chaarriau						
Rachel	Chaigeau						
Taylor	Chalmers						
Marie-Thérèse	Clerc						
Montserrat	Clerigue						

*First name, last name, and suffix (if applicable) are required and will appear in PubMed.

***First Name and Middle Initial(s)**	***Last Name**	***Suffix (eg, Jr, III)**	Academic Degrees	Institution	Location (city, state/province, country)	Role or Contribution, eg, chair, principal investigator	Group (if more than 1 Group listed in the byline) and/or Subgroup (eg, Steering Committee)
Emmanuel	Cognat						
Nina	Coll						
Lyduine E	Collij						
Peter	Connely						
Elodie	Cordier						
Corine	Costes						
Camille	Coulange						
Hélène	Courtemanche						
Eric	Creisson						
Charlotte	Crinquette						
Rosario	Cuevas						
Marie-Noëlle	Cufi						
Sophie	Dardenne						
Maria	de Arriba						
Casper	de Costa Luis						
Yvonne	de Gier						
Delphine	de Verbizier Lonjon						
Veronique	Dekker						
Bérengère	Dekyndt						
Xavier	Delbeuck						
Julien	Delrieu						
Jean-François	Demonet						
Vincent	Deramecourt						
Françoise	Desclaux						
Carlos	Diaz						
Susana	Diego						
Mehdi	Djafar						
Britta	Dölle						
Laura	Doull						
Laurence	Dricot						

Supplemental Online Content: Nonauthor Collaborators
*First name, last name, and suffix (if applicable) are required and will appear in PubMed.

***First Name and Middle Initial(s)**	***Last Name**	***Suffix (eg, Jr, III)**	Academic Degrees	Institution	Location (city, state/province, country)	Role or Contribution, eg, chair, principal investigator	Group (if more than 1 Group listed in the byline) and/or Subgroup (eg, Steering Committee)
Alexander	Drzezga						
Bruno	Dubois						
Julien	Dumont						
Jean	Dumur						
Julien	Dumurgier						
Martin	Dvorak						
Mirian	Ecay						
Paul	Edison						
Claus	Escher						
Ainara	Estanga						
Ester	Esteban						
Guy	Fanjaud						
Gill	Farrar						
Karine	Fauria						
Marta	Felez Sanchez						
Patrick	Feukam Talla						
Lisa	Ford						
Giovanni B	Frisoni						
David	Fuster						
Audrey	Gabelle						
Valentina	Garibotto						
Sinead	Gaubert						
Cédric	Gauci						
Christine	Geldhof						
Jean	Georges						
Joseph	Ghika						
Rossella	Gismondi						
Juan Domingo	Gispert						
Elena	González						
Valerie	Goovaerts						
Denis Mariano	Goulart						

Supplemental Online Content: Nonauthor Collaborators
*First name, last name, and suffix (if applicable) are required and will appear in PubMed.

***First Name and Middle Initial(s)**	***Last Name**	***Suffix (eg, Jr, III)**	Academic Degrees	Institution	Location (city, state/province, country)	Role or Contribution, eg, chair, principal investigator	Group (if more than 1 Group listed in the byline) and/or Subgroup (eg, Steering Committee)
Caroline	Grasselli						
Oriol	Grau-Rivera						
Katherine	Gray						
Martin	Greensmith						
Laure	Grozn						
Céline	Guillemaud						
Fiona	Gunn						
Prasad	Guntur Ramkumar						
Göran	Hagman						
Bernard	Hansseuw						
Fiona	Heeman						
Janine	Hendriks						
Jakob	Himmelmann						
Anne	Hitzel						
Florent	Hives						
Merle	Hoenig						
Claire	Hourrègue						
Justine	Hudson						
Jordi	Huguet						
Marta	Ibarria						
Ifrah	Iidow						
Sandrine	Indart						
Silvia	Ingala						
Adrian	Ivanoiu						
Charlotte	Jacquemont						
Vesna	Jelic						
Frank	Jessen						
Jieqing	Jiao						
Sara	Jofresa						
Cathrine	Jonsson						

Supplemental Online Content: Nonauthor Collaborators
*First name, last name, and suffix (if applicable) are required and will appear in PubMed.

***First Name and Middle Initial(s)**	***Last Name**	***Suffix (eg, Jr, III)**	Academic Degrees	Institution	Location (city, state/province, country)	Role or Contribution, eg, chair, principal investigator	Group (if more than 1 Group listed in the byline) and/or Subgroup (eg, Steering Committee)
Dzmitry	Kaliukhovich						
Silke	Kern						
Miia	Kivipelto						
Iva	Knezevic						
Grégory	Kuchcinski						
Manon	Laforce						
Asunción	Lafuente						
Françoise	Lala						
Adriaan	Lammertsma						
Michelle	Lax						
Thibaud	Lebouvier						
Ho-Yun	Lee						
Lean	Lee						
Annebet	Leeuwis						
Amandine	Lefort						
Jean-François	Legrand						
Mélanie	Leroy						
Constance	Lesoil Markowski						
Marcel	Levy						
Renaud	Lhommel						
Renaud	Lopes						
Isadora	Lopes Alves						
Luigi	Lorenzini						
Adrien	Lorette						
Emma	Luckett						
Marie	Lundin						
Marie-Anne	Mackowiak						
Vincent	Malotaux						
Richard	Manber						
Nikolay	Manyakov						

Supplemental Online Content: Nonauthor Collaborators
*First name, last name, and suffix (if applicable) are required and will appear in PubMed.

***First Name and Middle Initial(s)**	***Last Name**	***Suffix (eg, Jr, III)**	Academic Degrees	Institution	Location (city, state/province, country)	Role or Contribution, eg, chair, principal investigator	Group (if more than 1 Group listed in the byline) and/or Subgroup (eg, Steering Committee)
Pawel	Markiewicz						
Paula	Marne						
Marta	Marquié						
Elvira	Martín						
Joan	Martínez						
Pablo	Martinez Lage						
Sophie E	Mastenbroek						
Aurélien	Maureille						
Karen	Meersmans						
Anja	Mett						
Joseph	Milne						
Carolina	Minguillón						
Marc	Modat						
José Luis	Molinuevo						
Laura	Montrreal						
Christian	Moro						
Theresa	Müller						
Graciela	Muniz						
Henk Jan	Mutsarts						
Ted	Nilsson						
Aida	Ninerola						
Agneta	Nordberg						
Wilse	Novaes						
Joao	Nuno Carmelo Pires Silva						
Greg	Operto						
Adela	Orellana						
Pierre-Jean	Ousset						
Olivier	Outteryck						
Amandine	Pallardy						
Alessandro	Palombit						

*First name, last name, and suffix (if applicable) are required and will appear in PubMed.

***First Name and Middle Initial(s)**	***Last Name**	***Suffix (eg, Jr, III)**	Academic Degrees	Institution	Location (city, state/province, country)	Role or Contribution, eg, chair, principal investigator	Group (if more than 1 Group listed in the byline) and/or Subgroup (eg, Steering Committee)
Ana	Pancho						
Martin	Pappon						
Claire	Paquet						
Jérémie	Pariente						
Florence	Pasquier						
Pierre	Payoux						
Harry	Peaker						
Esther	Pelejà						
Delphine	Pennetier						
Alba	Pérez-Cordón						
Andrés	Perissinotti						
Matthieu Paul	Perrenoud						
Sandrine	Petit						
Grégory	Petyt						
Julia	Pfeil						
Blanche	Pirotte						
Sandra	Pla						
Sonia	Plaza Wuthrich						
Lea	Poitrine						
Marianne	Pollet						
Jean-Benoit	Poncelet						
John	Prior						
Jean-Pierre	Pruvo						
Pauline	Putallaz						
Mathieu	Queneau						
Lisa	Quenon						
Andreea	Rădoi						
Marie	Rafiq						
Fiona	Ramage						
Maribel	Ramis						
Michael	Reinwald						

Supplemental Online Content: Nonauthor Collaborators
*First name, last name, and suffix (if applicable) are required and will appear in PubMed.

***First Name and Middle Initial(s)**	***Last Name**	***Suffix (eg, Jr, III)**	Academic Degrees	Institution	Location (city, state/province, country)	Role or Contribution, eg, chair, principal investigator	Group (if more than 1 Group listed in the byline) and/or Subgroup (eg, Steering Committee)
Gonzalo	Rios						
Craig	Ritchie						
Elena	Rodriguez						
Adeline	Rollin						
Olivier	Rouaud						
Simona	Sacuiu						
Laure	Saint-Aubert						
Arianna	Sala						
Anne-Sophie	Salabert						
Jon	Saldias						
Gemma	Salvadó						
Angela	Sanabria						
Lena	Sannemann						
Nathalie	Sastre						
Daniela	Savina						
Irina	Savitcheva						
Jolien	Schaeverbeke						
Philip	Scheltens						
Carine	Schildermans						
Mark	Schmidt						
Michael	Schöll						
Jeroen	Schuermans						
Franck	Semah						
Mahnaz	Shekari						
Ingmar	Skoog						
Oscar	Sotolongo-Grau						
Andrew	Stephens						
Tiffany	Stewart						
Jennyfer	Stutzmann						
Murray	Tait						

Supplemental Online Content: Nonauthor Collaborators
*First name, last name, and suffix (if applicable) are required and will appear in PubMed.

***First Name and Middle Initial(s)**	***Last Name**	***Suffix (eg, Jr, III)**	Academic Degrees	Institution	Location (city, state/province, country)	Role or Contribution, eg, chair, principal investigator	Group (if more than 1 Group listed in the byline) and/or Subgroup (eg, Steering Committee)
Lluis	Tárraga						
Juan Pablo	Tartari						
Ann-christine	Tysen-backstrom						
Sergi	Valero						
David	Vallez Garcia						
Bart N M	van Berckel						
Martijn	van Essen						
Koen	Van Laere						
Jeroen	van Leur						
Ingrid S	van Maurik						
Rik	Vandenberghe						
Bruno	Vellas						
Jukka	Virolinen						
Pieter Jelle	Visser						
Zuzana	Walker						
Håkan	Walles						
Emilia	Wallin						
Grant	Whitelaw						
Catriona	Wimberley						
Zarni	Win						
Alle Meije	Wink						
Robin	Wolz						
John	Woodside						
Maqsood	Yaqub						
Anna	Zettergren						
Philip	Zeyen						
