# Supplementary material for: Analysis of Psychological Symptoms Following Disclosure of Amyloid–Positron Emission Tomography Imaging Results to Adults With Subjective Cognitive Decline
Source: JAMA Netw Open. 2023 Jan 13;6(1):e2250921. doi: 10.1001/jamanetworkopen.2022.50921 (PMC9857261; doi:10.1001/jamanetworkopen.2022.50921)
Supplement: Supplement 3. — Data Sharing Statement [file jamanetwopen-e2250921-s003.pdf]

Data Sharing Statement

Caprioglio. Analysis of Psychological Symptoms Following Disclosure of Amyloid-Positron Emission Tomography Imaging Results to Adults With Subjective Cognitive Decline. *JAMA Netw Open*. Published January 13, 2023. doi:10.1001/jamanetworkopen.2022.50921

Data
Data available: Yes
Data types: Deidentified participant data, Data dictionary
How to access data: Anonymized data collected for the study and additional documents (e.g. study protocol, informed consent form) may be made available to others upon request and after the approval of a proposal by the AMYPAD consortium.
When available: With publication

Supporting Documents
Document types: None

Additional Information
Who can access the data: Data will be made available to anyone requesting the data, upon request and after the approval of a proposal by the AMYPAD consortium.
Types of analyses: Data will be made available for any purpose, upon request and after the approval of a proposal by the AMYPAD consortium.
Mechanisms of data availability: Data will be made available upon request and after the approval of a proposal by the AMYPAD consortium.
